# Chaperone-Mediated Autophagy Upregulation Rescues Megalin Expression and Localization in Cystinotic Proximal Tubule Cells

**DOI:** 10.3389/fendo.2019.00021

**Published:** 2019-02-01

**Authors:** Jinzhong Zhang, Jing He, Jennifer L. Johnson, Farhana Rahman, Evripidis Gavathiotis, Ana Maria Cuervo, Sergio D. Catz

**Affiliations:** ^1^Department of Molecular Medicine, The Scripps Research Institute, La Jolla, CA, United States; ^2^Department of Biochemistry, Albert Einstein College of Medicine, Bronx, NY, United States; ^3^Department of Developmental and Molecular Biology, Albert Einstein College of Medicine, Bronx, NY, United States

**Keywords:** lysosomal storage disorder (LSD), fanconi syndrome, megalin, chaperone-mediated autophagy (CMA), cystinosis, vesicular trafficking, Rab 11 GTPase, LAMP2A

## Abstract

Cystinosis is a lysosomal storage disorder caused by defects in *CTNS*, the gene that encodes the lysosomal cystine transporter cystinosin. Patients with nephropathic cystinosis are characterized by endocrine defects, defective proximal tubule cell (PTC) function, the development of Fanconi syndrome and, eventually, end-stage renal disease. Kidney disease is developed despite the use of cysteamine, a drug that decreases lysosomal cystine overload but fails to correct overload-independent defects. Chaperone-mediated autophagy (CMA), a selective form of autophagy, is defective in cystinotic mouse fibroblasts, and treatment with cysteamine is unable to correct CMA defects *in vivo*, but whether the vesicular trafficking mechanisms that lead to defective CMA in cystinosis are manifested in human PTCs is not currently known and whether PTC-specific mechanisms are corrected upon CMA upregulation remains to be elucidated. Here, using CRISPR-Cas9 technology, we develop a new human PTC line with defective cystinosin expression (*CTNS*-KO PTCs). We show that the expression and localization of the CMA receptor, LAMP2A, is defective in *CTNS*-KO PTCs. The expression of the lipidated form of LC3B, a marker for another form of autophagy (macroautophagy), is decreased in *CTNS*-KO PTCs indicating decreased autophagosome numbers under basal conditions. However, the autophagic flux is functional, as measured by induction by starvation or by blockage using the v-ATPase inhibitor bafilomycin A, and by degradation of the macroautophagy substrate SQSTM1 under starvation and proteasome-inhibited conditions. Previous studies showed that LAMP2A accumulates in Rab11-positive vesicles in cystinotic cells. Here, we show defective Rab11 expression, localization and trafficking in *CTNS*-KO PTCs as determined by confocal microscopy, immunoblotting and TIRFM. We also show that both Rab11 expression and trafficking in cystinotic PTCs are rescued by the upregulation of CMA using small-molecule CMA activators. Cystinotic PTCs are characterized by PTC de-differentiation accompanied by loss of the endocytic receptor megalin, and megalin recycling is regulated by Rab11. Here we show that megalin plasma membrane localization is defective in *CTNS*-KO PTCs and its expression is rescued by treatment with CMA activators. Altogether, our data support that CMA upregulation has the potential to improve PTC function in cystinosis.

## Introduction

Cystinosis is a lysosomal storage disorder (LSD) caused by genetic defects in *CTNS* (*Ctns* in mouse), the gene that codes for the cystine transporter cystinosin. Increased levels of intra-lysosomal cystine (1) lead to cell malfunction in cystinosis. Tissue deterioration manifests in kidneys and eyes but also affects other organs including the liver, brain, and muscle (2). Kidney proximal tubule cells (PTCs) are the first cell type to be affected in nephropathic cystinosis, causing, in the long term, end-stage kidney disease. Patients with severe cystinosis require kidney transplants. Endocrine disorders are also common in cystinosis such as hypothyroidism, growth retardation, and hypogonadism (3). Hypothyroidism is the most frequently reported endocrine manifestation of the disease (4). Altered thyroglobulin biosynthesis associated with endoplasmic reticulum stress is the cause of this manifestation. Cystinotic patients also suffer from insulin-dependent diabetes (5), which contributes other complications including muscle (6) and bone (7) alterations that are pathognomonic of the disease.

The current treatment for patients with cystinosis is cysteamine which reduces intra-lysosomal cystine, conjugates, and transports cysteine out of the lysosome through the exporter PQLC2 (8). Despite the efficiency of cysteamine in retarding the rate of renal deterioration and improving linear growth in children with cystinosis (9), cell malfunction, tissue failure, progressive renal disease, endocrine complications, and muscle abnormalities still occur (10), suggesting that cystine accumulation is not the only cause for all the defects observed in cystinosis (10, 11). Thus, to improve treatment of this LSD, it is crucial to understand the defective molecular mechanisms that lead to the various tissues dysfunction and injury. In order to understand these mechanisms, it is essential to develop and characterize models of the disease. To this end, the establishment of new cellular models of cystinotic proximal tubule cells, with defined genotypic and phenotypic characteristics, is essential to study disease-relevant mechanisms, to develop knowledge and to implement novel strategies for treating renal disease progression in this devastating disease.

Chaperone mediated autophagy (CMA) is a selective form of autophagy that contributes to proteostasis in several physiological and pathological conditions (12). CMA consists of the internalization of selected cytosolic substrates into the lysosome by a mechanism that includes: Recognition of a pentapeptide-like KFERQ in the substrate by the chaperone hsc70; substrate presentation by the chaperone to the receptor LAMP2A; receptor multimerization and protein internalization for degradation in the lysosome, assisted by a lumenal form of hsc70 (13). LAMP2A the only known lysosomal receptor for CMA, shows defective localization and impaired function in cystinosis (14, 15). Defects in CMA in cystinosis lead to the cytosolic accumulation of CMA substrates and are proposed to contribute to the pathological processes of the disease that are cysteamine treatment-insensitive (14). However, the specific CMA mechanism(s) that are defective in cystinotic proximal tubule cells are currently unknown and the impact of CMA upregulation on PTC function requires further analysis. Under oxidative stress CMA is activated. This activation correlates with increased expression levels of the lysosomal lumenal chaperone protein hsc70 (required for substrate uptake), and also correlates with a selective increase of the expression of the CMA receptor LAMP2A at the lysosomal membrane, leading to higher rates of CMA (16). However, despite the observations that cystinosis is associated with increased oxidative stress and that cystinotic patients have high serum levels of oxidative stress markers (11), cystinotic cells are actually susceptible to oxidative stress, most likely caused by downregulation of CMA. Remarkably, CMA induction by pharmacological enhancers protects cystinotic cells from the increased susceptibility to oxidative stress and reconstitutes the resistant levels observed in wild type cells, an effect dependent on LAMP2A expression and its lysosomal membrane localization (15). It then becomes clear that the correct lysosomal localization of LAMP2A is necessary to maintain cellular homeostasis in cystinosis. However, the mechanisms that mediate lysosomal localization of LAMP2A are not well-understood and the possible consequences of downregulated CMA in cystinotic PTCs is unknown.

In cystinosis, cystine accumulation induces apical PTC dedifferentiation (17). PTCs, which play a central role in maintaining homeostasis by mediating reabsorption of electrolytes and nutrients in the renal tube, rely on specialized apical receptors that control the internalization of specific substrates. In particular, megalin (gp330, LRP-2), a member of the low-density lipoprotein receptor family, is expressed in proximal tubule epithelial cells, and together with cubilin, mediates the endocytosis of an extensive number of diverse ligands including lipoproteins, vitamin-binding proteins, hormones, and enzymes (18). Based on their function as non-specific protein reabsorption molecules, megalin and cubilin are considered to operate as scavenger receptors (19). Megalin's apical localization in PTCs is mediated by fast-recycling mechanisms from apical recycling endosomes, a mechanism regulated by the small GTPase Rab11 (18). Fast megalin recycling allows for its reutilization and delays its degradation (18). A possible role for CMA in the regulation of megalin expression and localization is currently unknown. However, the role of CMA in the control of trafficking mechanisms that are affected in cystinotic fibroblasts, as previously described by our group (14), suggests that autophagy and, in particular CMA, may be an important determinant in the control of megalin functions through the regulation of vesicular transport.

In this work, we developed and characterized human cystinotic proximal tubule cells (*CTNS*-KO PTCs) and demonstrated that these cells are characterized by CMA defects that affect vesicular trafficking mechanisms regulating megalin localization.

## Materials and Methods

### Generation of Cystinosin Knockout Human Proximal Tubule Cell Line Using CRISPR/Cas9

To generate *CTNS* knockout human proximal tubule cells, Cas9 nickase was used along with a pair of gRNAs targeting exon 4 of the human *CTNS* gene. gRNAs were designed using the online tool (http://crispr.mit.edu/). Each gRNA was sub-cloned into pSpCas9n(BB)-2A-GFP(PX461) (Addgene), two pSpCas9n(BB)-2A-GFP(PX461)-*CTNS* gRNA constructs were then transfected into a model of human proximal tubule cell (HK-2 cells) at a 1:1 ratio using Lipofectamine LTX (Thermo Fisher Scientific). After 48 h, GFP-positive cells were sorted by FACS, seeded into plates and grown into single colonies. Single colonies were then expanded, the junction sequence was amplified by PCR, sub-cloned into pBluescript and sequenced to identify the variety of insertions and deletions (indels) at targeting sites. As a result, we obtained different indels in two alleles. One of the modifications has a 37 bp insertion, which abolishes the splicing site of the exon 4 at its 3′ end. The other allele has a 17 bp deletion, causing an amino acid shift, which led to a 52-amino acid product.

### Constructs and Transfections

The GFP-Rab11 construct was obtained from Addgene. Cells were transfected with this construct using Lipofectamine® 2000 (Thermo Fisher Scientific) following the manufacturer's instructions.

### Gel Electrophoresis, Immunoblotting, and Antibodies

Cells were lysed in RIPA lysis buffer in the presence of protease-inhibitors (Roche). Following electrophoresis using NuPAGE 4–12% gels (Thermo Fisher Scientific), proteins were transferred onto 0.45 μm nitrocellulose membranes and the membranes were incubated overnight at 4°C with the indicated primary antibodies, followed by incubation with HRP-conjugated secondary antibodies. The following antibodies were used in this study: mouse anti-LAMP1 (Santa Cruz, sc-20011), rabbit anti-LAMP2A (Abcam, ab18528), rabbit anti-actin (Sigma, A2066), mouse anti-Rab11 (Thermo Fisher Scientific, MA1-24919), rabbit anti-SQSTM1/p62 (Cell Signaling, 5114), rabbit anti-LC3B (Cell Signaling, 2775), and goat anti-Megalin (Santa Cruz, sc-16476).

### Immunofluorescence and Confocal Microscopy Analysis

Cells were seeded on a 4-chamber 35 mm-glass bottom dish (in vitro Scientific). Where indicated, cells were treated with the indicated treatments, then fixed with 3.7% paraformaldehyde for 15 min and blocked with 1% BSA in PBS for 1 h. Samples were labeled with the indicated primary antibodies overnight at 4°C in the presence of 0.01% saponin and 1% BSA. Samples were washed 3 times and subsequently incubated with the appropriate combinations of Alexa Fluor (488 or 594)-conjugated anti-rabbit, anti-rat, or anti-mouse secondary antibodies (Thermo Fisher scientific). Samples were analyzed with a Zeiss LSM 710 laser scanning confocal microscope (LSCM) attached to a Zeiss Observer Z1 microscope, using a 63 × oil Plan Apo, 1.4 NA objective. Images were collected using ZEN-LSM software keeping the laser power and gain constant during all acquisitions for comparative analysis of wild type and knockout cells or treated with vehicle or various treatments. Images were then processed using Image*J* (National Institutes of Health, Bethesda, MD) and Photoshop CS4 (Adobe). The fluorescence intensities were quantified using the Image*J* software.

### Total Internal Reflection Fluorescence (TIRF) Microscopy

Pseudo-TIRF microscopy analyses were performed using a 100X 1.45 numerical aperture TIRF objective (Nikon) on a Nikon TE2000U microscope custom modified with a TIRF illumination module as described (20). Images were acquired on a 16-bit, cooled charge-coupled device camera (Hamamatsu) controlled through NIS-Elements software. For live experiments, the images were recorded using 300–500 ms exposures depending on the fluorescence intensity of the sample.

### Cystine Accumulation

Cystine levels were measured by mass spectrometry at the Biochemical Genetics Laboratory, University of California, San Diego as described previously (20).

### Starvation Protocols

For studies of macroautophagy, cells were briefly washed in serum-free DMEM (containing 1x amino acids), media was aspirated, and fresh serum-free DMEM was added followed by 5 h incubation at 37°C, in the presence or absence of lysosomal inhibitor Bafilomycin A (LC laboratories, 100 nM) or proteasome inhibitor Clasto-Lactacystin β-lactone (Cayman Chemical, 1 uM), as described previously (14).

### PCR and RT-PCR Analysis

For RT-PCR analysis, RNA was isolated from wild-type or *Ctns*^−/−^ mouse fibroblasts using the RNeasy mini-kit for RNA purification (Qiagen). A total of 100 ng of RNA for each cell line was reverse-transcribed (RT) using iScript cDNA synthesis kit (Bio-Rad). PCR analysis was then performed using Taq DNA Polymerase(Thermo Fisher Scientific, 18038042), with the following primer mixes: human *CTNS* forward, TCCTCCTGTCGTAAAGCTGGA, and human *CTNS* reverse, GCCGGTCTGATTGGAGTGAT.

### Statistical Analysis

Data are presented as mean, and error bars correspond to standard errors of the means (SEMs) unless otherwise indicated. Statistical significance was determined using the pair or unpaired Student's *t*-test or one-way ANOVA, Tukey's multiple comparisons test for multiple groups using either Excel software or GraphPad Prism (version 4) software.

## Results

### Generation and Characterization of Cystinotic Human Proximal Tubule Cells (PTCs)

**Figure 1:**
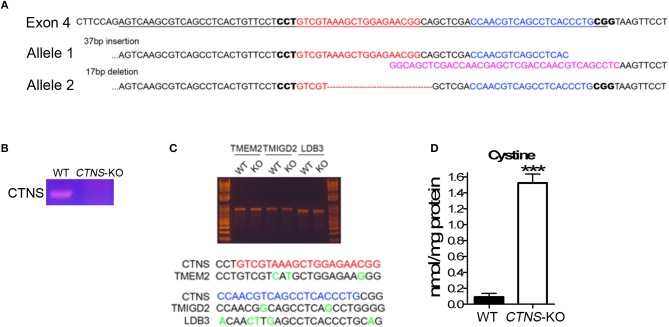
Generation of cystinosin knockout human proximal tubule cell line using CRISPR/Cas9. **(A)** Human *CTNS* genomic sequence; underlined sequences indicate exon 4, gRNA sequences were marked as red and blue, and PAM sequences are in bold. The allele 1 has 37bp insertion which was marked in pink; allele 2 has 17 bp deletion which was marked as a red dashed line. **(B)** PCR analysis of *CTNS* gene expression in WT and *CTNS*-KO HK-2 cells. **(C)** Surveyor assay of three potential off-target sites that localize in TMEM2, TMIGD2, and LDB3 genes. **(D)** Cystine levels in WT and *CTNS*-KO HK-2 cells were determined by mass spectrometry. The data are represented as mean ± SEM from three independent experiments. ^***^p < 0.001.

Proximal tubule cells (PTCs) are the most affected cells in cystinosis and are characterized by the accumulation of cystine crystals, as well as by PTC de-differentiation accompanied by loss of the endocytic receptors megalin and cubilin (17). In cystinotic fibroblasts, the cystinosin deficiency leads to defects in autophagic pathways caused by defective intracellular trafficking of the CMA receptor LAMP2A (14). In cystinotic kidneys, the accumulation of substrates suggests defective CMA but whether LAMP2A function is defective in *CTNS*-KO PTCs and whether CMA upregulation improves PTC function are both currently unknown. To study the mechanisms of vesicular trafficking and CMA regulation in *CTNS*-KO PTCs, we developed human PTCs deficient in cystinosin using CRISPR-Cas9 technology. To this end, we used the human PTC cell line HK-2, which has been largely demonstrated to be an authentic PTC model (21). The strategy to design and generate *CTNS*-KO PTCs is described in Figure 1. Two gRNAs targeting Human *CTNS* exon 4 were designed and are shown in Figure 1A. The gRNA1 targeted one of the alleles, generated a 37 bp insertion, destroyed the splicing site, and was predicted to generate a 149aa product. The gRNA2, targeted the second allele to generate a 17 bp deletion, causing a shift leading to a theoretical 52aa product (Figure 1A). Next, the *CTNS*-KO PTCs were analyzed for the expression of *CTNS* mRNA by PCR (Figure 1B). Using a 5′ primer that sits just upstream of the mutation site and a downstream reverse primer, we show that wild type *CTNS* mRNA is not expressed in *CTNS*-targeted (*CTNS*-KO) cells. Although the mutations deplete expression of *CTNS*, aberrant *CTNS* mRNA could still be detected, at least partially (not shown). Possible off-target effects of CRISPR/Cas9 were also analyzed. Thus, three potential off-target loci were amplified by PCR followed with Surveyor assay and Sanger sequencing. Our data show that these three loci are intact (Figure 1C). For confirmation of cystinosin activity deficiency, we measured cystine accumulation using mass spectrometry analysis of cystine content. In Figure 1D, we show that cystine levels are barely detectable in wild type cells but cystine is significantly increased in *CTNS*-KO cells. Thus, *CTNS*-KO PTCs accumulate cystine at levels that are similar to those observed in cystinotic cells from the *Ctns*^−/−^ mouse (14).

### Decreased Expression and Mislocalization of LAMP2A in *CTNS*-KO PTCs

**Figure 2:**
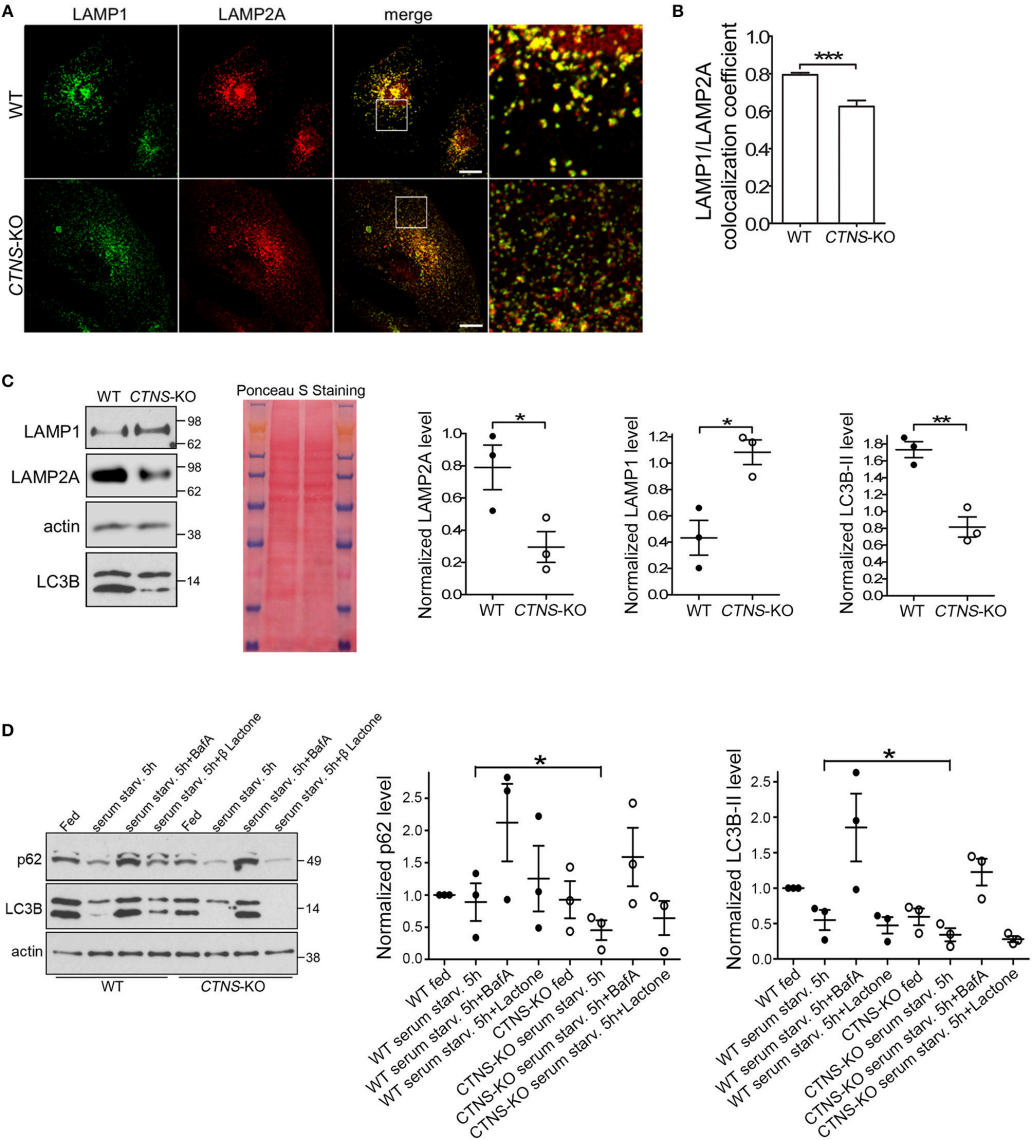
Mislocalization and decreased expression of LAMP2A in *CTNS*-KO PTCs. **(A,B)** Confocal microscopy analysis and quantification of the distribution of endogenous LAMP1 and LAMP2A in WT and *CTN*S-KO PTCs. Scale bar, 10 μm. mean ± SEM. ^***^p < 0.001, Student's *t*-test. **(C)** Western blot analysis of LAMP1, LAMP2A, and LC3B expression levels in WT and *CTN*S-KO PTCs. Left panel, Representative immunoblots. Ponceaus S staining is shown for equal loading. Middle and right panels, Quantification of 3 independent experiments. The individual symbols correspond to independent biological replicates from 3 independent experiments. The bars represent the mean ± SEM. ^*^p < 0.05; ^**^p < 0.01, Student's *t*-test. **(D)** SQSTM1/p62 and LC3B-II levels in WT and *CTN*S-KO PTCs were analyzed by Western blot under fed and serum starvation conditions, in the absence or presence of 100 nM bafilomycin A (BafA) or 1 μM Clasto-Lactacystin β-lactone (proteasome inhibitor) for 5 h. Left panel, Representative immunoblots. Middle and right panel, Quantification of the expression levels of SQSTM1 (p62) and LC3B-II, respectively. Each value has been normalized to the actin expression level in the same sample. Results are expressed relatively to the wild type fed condition. The individual symbols correspond to independent biological replicates from 3 independent experiments. The bars represent the mean ± SEM. ^*^p < 0.05.

To study possible CMA defective mechanisms in *CTNS*-KO PTCs, and because previous studies from our laboratory suggested that the CMA receptor LAMP2A is mislocalized in cystinosis, we first analyzed the subcellular distribution of LAMP2A in wild type and *CTNS*-KO PTCs. In Figures 2A,B, we show that while LAMP2A shows high colocalization with LAMP1 in wild type cells, *CTNS*-KO PTCs have a subpopulation of LAMP2A-positive vesicles that does not overlap with the distribution of LAMP1. This is in agreement with previous studies from our laboratory showing that intermediate vesicles containing LAMP2A are mislocalized and have defective trafficking in cystinotic fibroblasts (15). Further, to better understand whether the mislocalization of LAMP2A affects protein stability, we determined the levels of expression of LAMP2A in *CTNS*-KO PTCs. Interestingly, LAMP2A expression was decreased in *CTNS*-KO PTCs, suggesting either downregulation at the transcriptional level or increased degradation (Figure 2C). LAMP2A-decreased expression was specific since LAMP1 expression is elevated in *CTNS*-KO PTCs (Figure 2C). These data recapitulate the defective phenotypes observed in *Ctns*^−/−^ fibroblasts (14, 20) and thus confirmed that *CTNS*-KO PTCs constitute a valid cystinotic model.

Next, to determine whether changes in CMA are accompanied by defects in macroautophagy, we analyzed the expression levels of the lipidated form of LC3B (LC3B-II) by Western blot. In Figure 2C, we show that LC3B-II expression is decreased in *CTNS*-KO PTCs.

The autophagic flux in *CTNS*-KO PTCs was analyzed using well-established biochemical methods. Wild type and *CTNS*-KO PTCs were exposed to fed, serum starvation and autophagy-blocking conditions, using bafilomycin to inhibit the fusion of autophagosomes with lysosomes. In Figure 2D, we show that despite having decreased number of autophagosomes, *CTNS*-KO PTCs respond to starvation by degrading LC3B, indicating that macroautophagy is inducible by starvation in these cells. This was also confirmed by the decrease in protein levels of the autophagy substrate SQSTM1/p62 in these cells after starvation. Treatment with the proteasome inhibitor β-lactone did not affect protein degradation (Figure 2D), indicating that SQSTM1 is degraded mainly through macroautophagy in these cells. Increased LC3B levels after treatment with bafilomycin confirmed that autophagosome formation is functional in *CTNS*-KO PTCs and that the reduced LC3B levels together with reduced SQSTM1 levels are indicative of accelerated clearance rather than reduced autophagosome biogenesis. This is in agreement with previous studies showing increased autophagic flux in cystinotic fibroblasts, albeit with accumulation of autophagosomes (14), and goes hand-in-hand with previous studies showing upregulation of macroautophagy as a compensatory mechanism in response to reduced CMA. This suggests that *CTNS*-KO PTCs have a dysregulated macroautophagy phenotype, similar to other cystinotic cellular systems.

**Figure 3:**
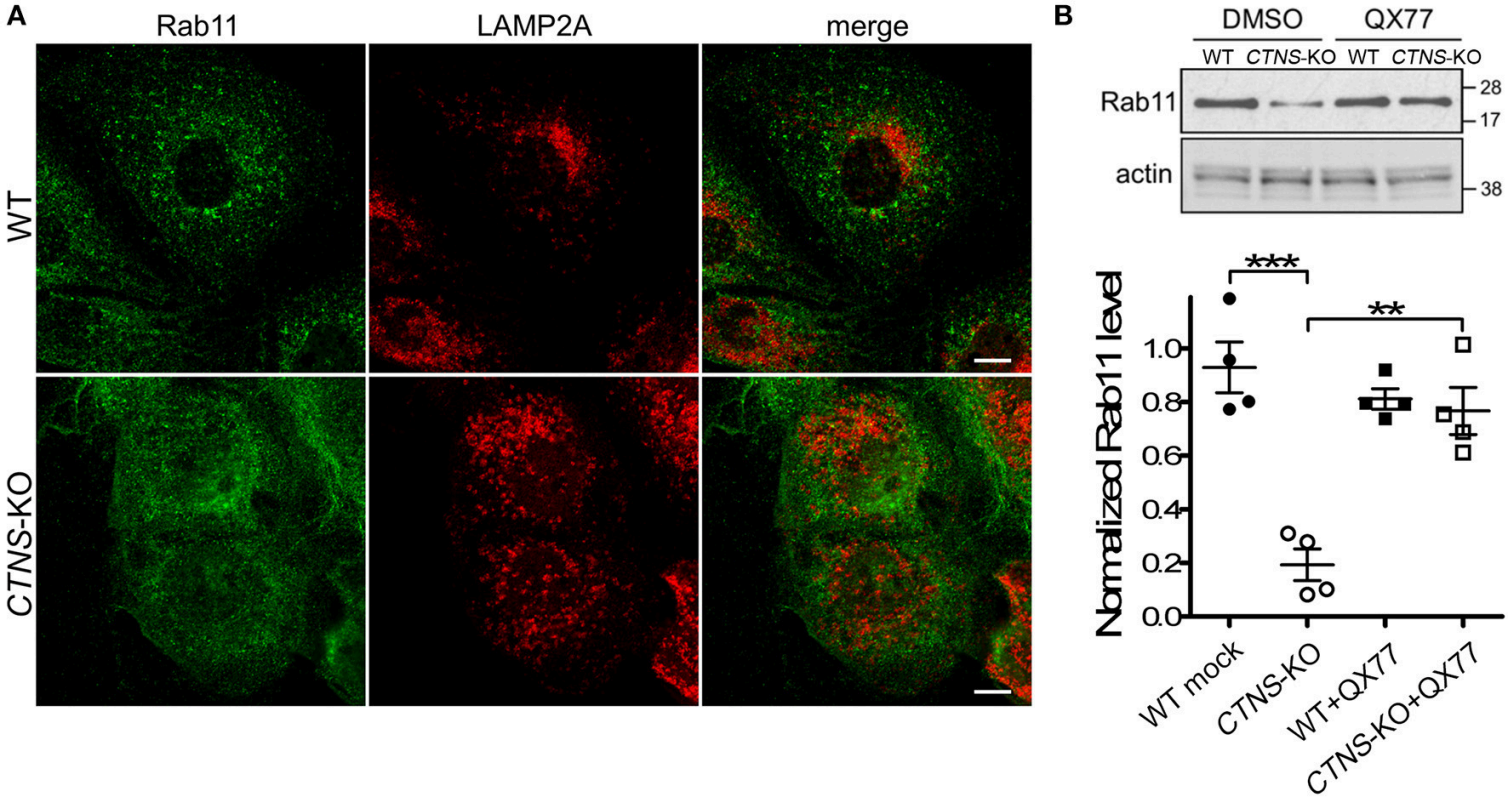
Rab11 is down-regulated and mislocalized in *CTNS*-KO PTCs. **(A)** Confocal microscopy analysis of the distribution of endogenous Rab11 and LAMP2A in WT and *CTN*S-KO PTCs. Scale bar, 10 μm. **(B)** WT and *CTN*S-KO PTCs were treated with DMSO or 20 μM QX77 for 48 h, and Rab11 expression levels were analyzed by Western blot. Quantitative analysis of Rab11 expression levels. The individual symbols correspond to independent biological replicates from 4 independent experiments. The expression level of Rab11 was normalized to actin in each sample. The bars represent the mean ± SEM. ^**^p < 0.01, and ^***^p < 0.001, Student's *t*-test.

We have previously shown that LAMP2A localizes at Rab11-positive vesicles in cystinotic cells (14), suggesting that Rab11-positive carrier vesicles may be responsible for trafficking of LAMP2A to the lysosome. Here, we next analyzed the distribution of endogenous Rab11 in *CTNS*-KO PTCs and found that Rab11 is mislocalized in these cells. In particular, we observed that the distribution of Rab11-positive vesicles is defective in *CTNS*-KO cells, showing a homogeneous distribution throughout the cystinotic cells as opposed to the more perinuclear distribution of Rab11 puncta observed in wild type cells (Figure 3A). This suggests defective trafficking of Rab11 vesicles in *CTNS*-KO cells. Next, we analyzed the expression levels of endogenous Rab11 and found that Rab11 is significantly downregulated in *CTNS*-KO PTCs (Figure 3B). Because LAMP2A mislocalization suggests defective CMA and as CMA upregulation may have a direct effect on the expression levels of trafficking associated proteins (15), we next studied the effect of CMA enhancers on Rab11 expression in *CTNS*-KO cells. To this end, we treated wild type and *CTNS*-KO PTCs with the CMA enhancer QX77, a small molecule known to dysregulate the repressive function of retinoic acid receptor-alpha over the CMA machinery (15, 22). We found that treatment with this CMA enhancer significantly increased Rab11 expression in *CTNS*-KO PTCs, bringing the expression of this small GTPase to wild type levels (Figure 3B).

**Figure 4:**
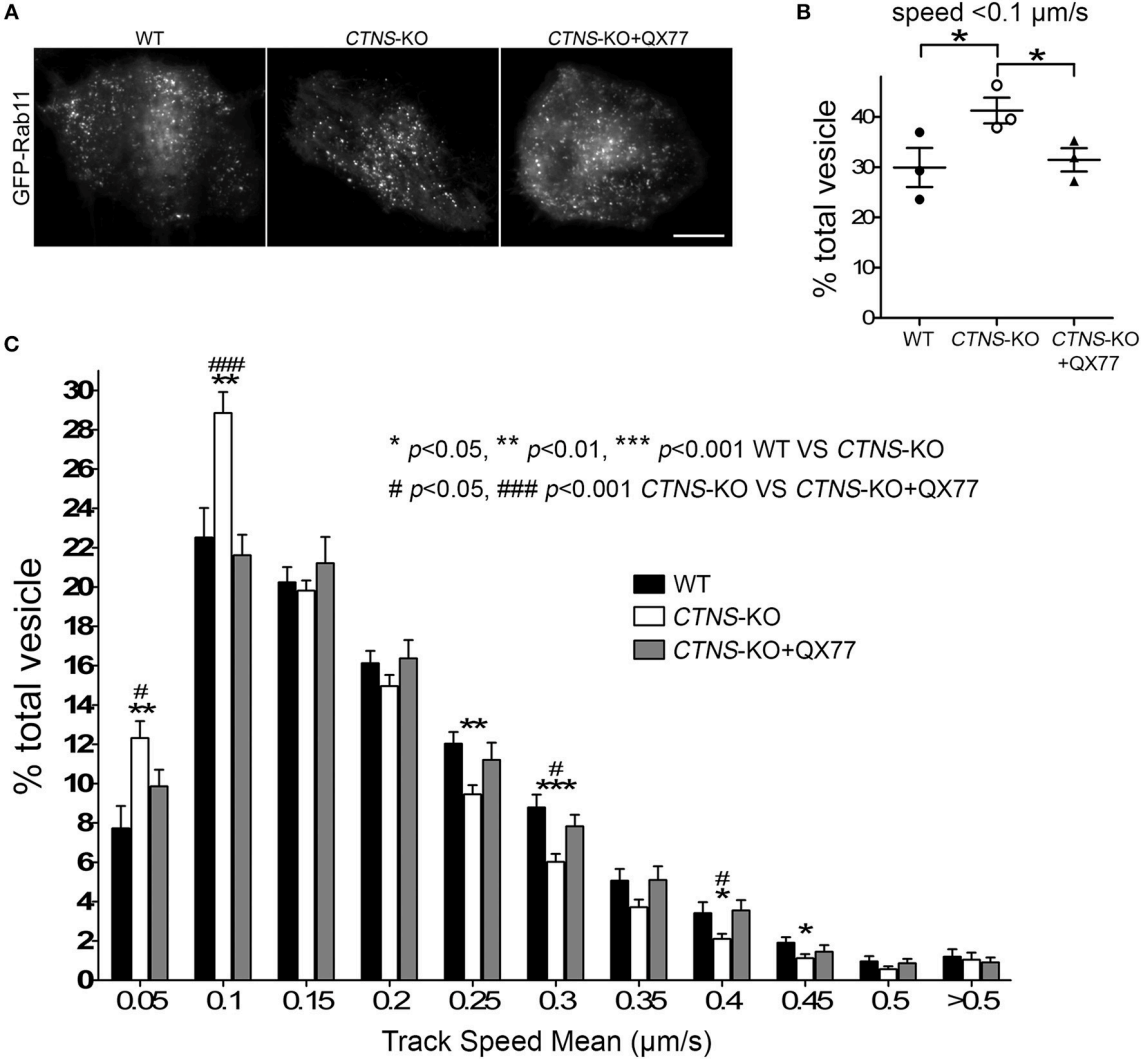
Rab11 trafficking is impaired in *CTNS*-KO PTCs and is enhanced by CMA activation. **(A)** Representative images of GFP-Rab11 in WT, *CTN*S-KO PTCs, and *CTN*S-KO PTCs treated with QX77. Scale bar, 10 μm. **(B,C)** Quantitative analysis of the trafficking of GFP-Rab11 in WT, *CTN*S-KO PTCs, and *CTN*S-KO PTCs treated with 20 μM QX77 for 72 h. **(B)** Quantitative analysis of the numbers of vesicles with decreased motility (speed < 0.1 μm/s) in wild type, *CTN*S-KO PTCs and *CTN*S-KO PTCs treated with QX77. The individual symbols correspond to independent biological replicates from 3 independent experiments. The bars indicate the mean ± SEM. ^*^p < 0.05. **(C)** Histograms represent the speeds of GFP-Rab11-containing organelles. The speeds for the independent vesicles were binned in 0.05 μm/s increments and plotted as a percentage of total vesicles for a given cell. Results are represented as mean ± SEM from at least 22 cells from 3 independent experiments. The statistically significant differences between the groups are indicated in the figure. Student's *t*-test.

Next, to determine whether the mislocalization of Rab11 was caused by defective trafficking, we transfected wild type and *CTNS*-KO cells with the recycling endosome trafficking reporter, GFP-Rab11 (Figure 4A), and measured vesicular transport using Total Internal Reflection Fluorescence microscopic analysis. In Figure 4B, we show that cystinotic PTCs have defective Rab11 trafficking characterized by significant decrease in Rab11-positive vesicle movement. Thus, *CTNS*-KO cells showed increased numbers of vesicles moving at very low speed or not moving at all (Figure 4B), with a concomitant decrease of faster-moving Rab11-positive vesicles (Figure 4C). Interestingly, treatment with the CMA enhancer QX77 rescued the defective trafficking phenotype in *CTNS*-KO PTCs thus favoring a net and significant increment in Rab11 transport (Figures 4B,C and Movies 1–3).

### Upregulation of Chaperone-Mediated Autophagy Increases Megalin Localization at the Plasma Membrane in Cystinotic PTCs

**Figure 5:**
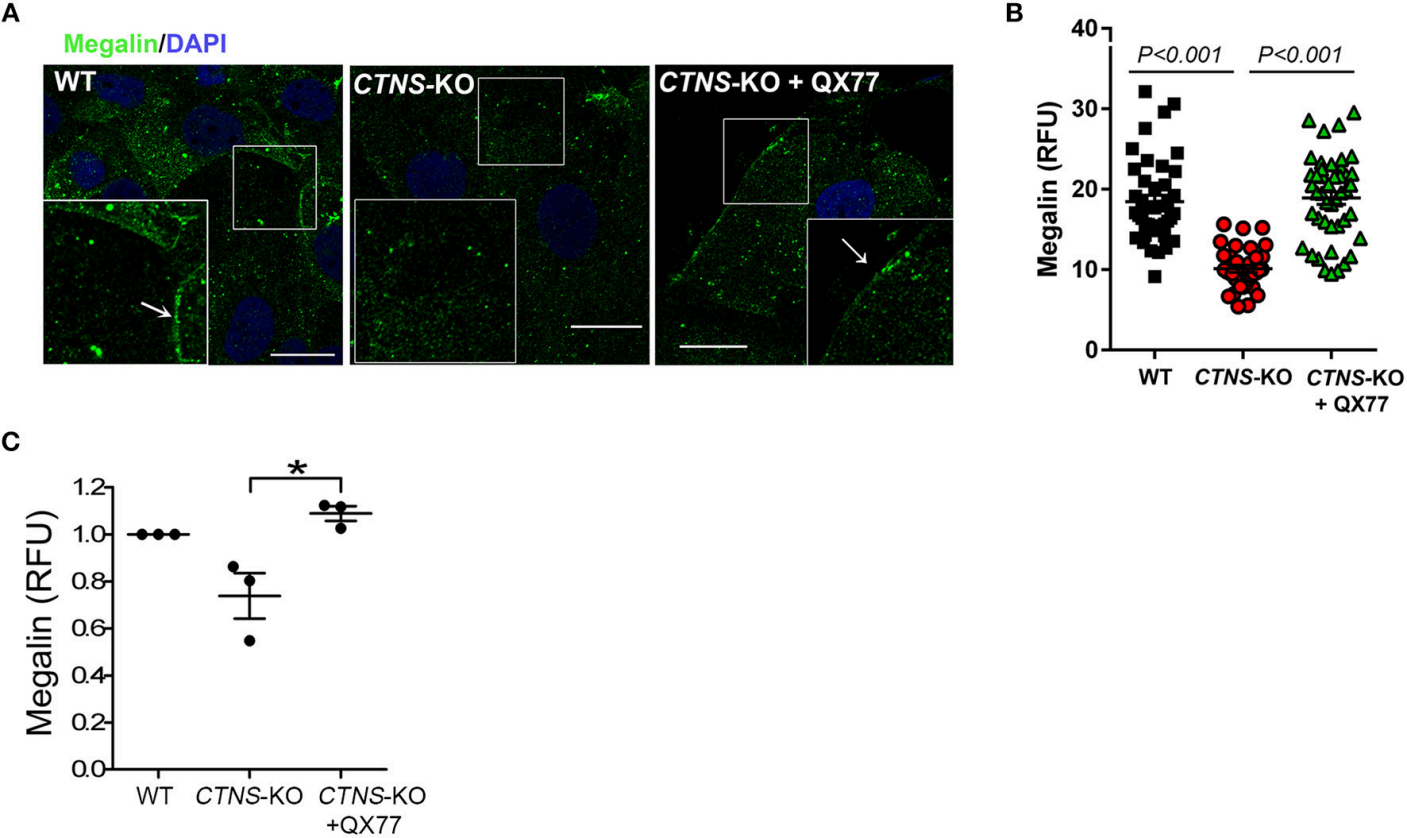
Decreased membrane localization and expression of megalin in *CTNS*-KO PTCs is rescued by CMA activation. **(A)** Confocal microscopy analysis of the distribution of endogenous megalin. WT, *CTNS*-KO PTCs, and *CTNS*-KO PTCs treated with 20 μM QX77 or vehicle for 72 h were fixed and endogenous megalin was detected by immunofluorescence (green) and nuclei stained with DAPI (blue). The arrows indicate plasma membrane localization of megalin. Scale bar, 10 μm. **(B)** Megalin expression was quantified by analysis of the mean fluorescence intensity of megalin in each cell using Image*J*. Each symbol represents a cell. The data are from one experiment, and are representative of three independent experiments with the same result. **(C)** Megalin expression was analyzed as in B. The individual symbols correspond to independent biological replicates from 3 independent experiments. The bars indicate the mean ± SEM. The data are normalized to the values observed in wild type cells. Significant differences between *CTNS*-KO untreated and QX77-treated *CTNS*-KO PTCs were calculated using the Student's *t*-test. ^*^p < 0.05.

Megalin, an endocytosis receptor located apically in PTCs and other epithelial cells including endocrine glands, the lung and the brain, plays a fundamental role in PTC function by facilitating the uptake of plasma solutes for ultrafiltration. Cystinotic PTCs have progressive loss of megalin expression and other apical receptors (17), and megalin-KO mice are characterized by urinary loss of ultra-filtrated plasma proteins (23). The apical distribution of megalin in PTCs is proposed to be mediated by the trafficking of apical recycling endosomes, regulated by the small GTPase Rab11 (18). Because Rab11 trafficking is defective in *CTNS*-KO PTCs, we next investigated the expression and localization of endogenous megalin in *CTNS*-KO PTCs. In Figure 5A, we show that *CTNS*-KO PTCs are characterized by decreased plasma membrane localization of megalin. Further, because treatment with CMA enhancers has a direct impact on Rab11 trafficking in cystinotic cells, we studied whether the upregulation of CMA has a beneficial effect on megalin localization. To this end, we treated *CTNS*-KO PTCs with the CMA activator QX77 (20 μM) for 72 h and determined the plasma membrane distribution of megalin in these cells. In Figure 5A, we show that CMA upregulation significantly increases megalin localization at the plasma membrane in *CTNS*-KO PTCs, indicating that upregulation of CMA has a direct positive implication on megalin distribution, likely affecting PTC function. Finally, because recycling through the plasma membrane is known to stabilize megalin and prevent its degradation (18), we next analyzed the effect of CMA enhancers on megalin expression. In Figures 5B,C, we show that megalin expression is decreased in *CTNS*-KO PTCs. We also show that treatment with the CMA enhancer, QX77, induces the significant upregulation of megalin expression in cystinotic PTCs.

## Discussion

Proximal tubule cell dedifferentiation is a hallmark of nephropathic cystinosis (17). One of the most important changes underwent by PTCs in cystinosis is the loss of megalin apical distribution which is accompanied by a net increase of protein and electrolyte loss (17). This progressive loss of the expression of megalin and other endocytic receptors, including the glucose and sodium transporter SGC2 and phosphate transporters, is proposed to account for apical dedifferentiation leading to the development of Fanconi syndrome, which is not corrected by cysteamine treatment in cystinotic patients. Megalin is a fast recycling receptor and has been proposed to define the apical recycling pathway of epithelial cells. Its localization and recycling at the apical membrane depends on the function of Rab11 (18). In this work, using newly generated cystinotic PTCs, we show that Rab11 expression and trafficking is defective in *CTNS*-KO cells. We also demonstrate that both the impaired Rab11 trafficking and the defective localization of the CMA receptor LAMP2A at lysosomes are corrected by treatment with CMA enhancers, thus linking CMA defects to PTC disfunction and de-differentiation in cystinosis.

Rab GTPases and their effectors are determinants of membrane identity and master regulators of vesicular function. Decreased expression or defective functions of these small GTPases are associated with specific defects in vesicular transport. Cystinotic cells are characterized by defective endolysosomal transport, while the induction of vesicular trafficking mechanisms through the upregulation of Rab27a or Rab7 improves vesicular trafficking in cystinosis (20). Furthermore, a Rab11-specific defect in cystinotic cells was recently described (15). In particular, the CMA receptor LAMP2A shows an anomalous distribution in mouse cystinotic fibroblasts, where it localizes to Rab11-positive carrier vesicles instead of reaching the lysosomes (14). LAMP2A mislocalization was directly associated with defects in CMA, observed both in isolated cystinotic fibroblasts and, *in vivo*, in cystinotic mice (14, 15). Constitutively active Rab11 partially rescues the localization of LAMP2A at the lysosomal membrane (15). It is current knowledge that not all defects in cystinosis are corrected by cysteamine, that Fanconi syndrome is developed before the apparition of cystine crystals and independently of early cysteamine treatment, suggesting that CMA defects occur independently of lysosomal overload. Because the defects in CMA function are not repaired by treatment with cysteamine, the only lysosomal depletion therapy currently available for cystinotic patients, CMA function acquires important clinical significance. In this context, the observations that CMA upregulation, through treatment of proximal tubule cells with novel CMA enhancers, upregulates Rab11 expression, corrects Rab11 trafficking and corrects LAMP2A localization in PTCs is highly significant. Of note, although LAMP2A is cleaved by lysosomal cathepsin A (24) and lysosomal protease inhibitors increase the amount of lysosomal membrane-localized LAMP2A in cystinosis (14), it is not yet clear whether this mechanism of LAMP2A upregulation may have direct implications in proximal tubule cell function.

How defective CMA affects PTC function in cystinosis is currently unknown but since dysfunction of CMA has been implicated in PTC hypertrophy (25), it is expected that CMA upregulation would be beneficial in cystinotic patients. Kidney proximal epithelial cells are characterized by low levels of macroautophagy, but high basal levels of CMA activity. In PTCs, CMA has been shown to regulate the protein levels of important transcription factors that control PTC differentiation, including Pax2 (25). In mouse cystinotic PTCs *in vivo*, the accumulation of CMA substrates (including GAPDH) is caused by mislocalization of LAMP2A (15), suggesting that CMA may directly affect PTC function in this lysosomal disorder and that LAMP2A trafficking may be affected in these cells. Despite this knowledge, a direct impact of CMA deficiency on PTC function in cystinosis has not been demonstrated thus far and the mechanisms underlying PTC defects caused by defective CMA in cystinosis remain unknown. Here, the observation that CMA enhancers upregulate Rab11 trafficking in cystinotic PTCs is highly significant, not only because Rab11 is able to re-localize LAMP2A at the lysosomal membrane (15) but also because Rab11 upregulation has direct consequences on plasma membrane expression of the endocytic receptor megalin, which as mentioned above, is essential for substrate resorption from the ultrafiltrate in the proximal tubule. Thus, it is expected that CMA enhancers would have a direct impact on cystinotic PTC function and potentially on the reduction of some of the symptoms associated with Fanconi syndrome. Whether CMA enhancers increase upregulation of other apical receptors in cystinosis is currently unknown, but because of their effect on Rab11 trafficking and the function of Rab11 as a recycling endosome regulator, it is likely that the effects of CMA enhancers go beyond the benefits mediated by megalin upregulation.

**Figure 6:**
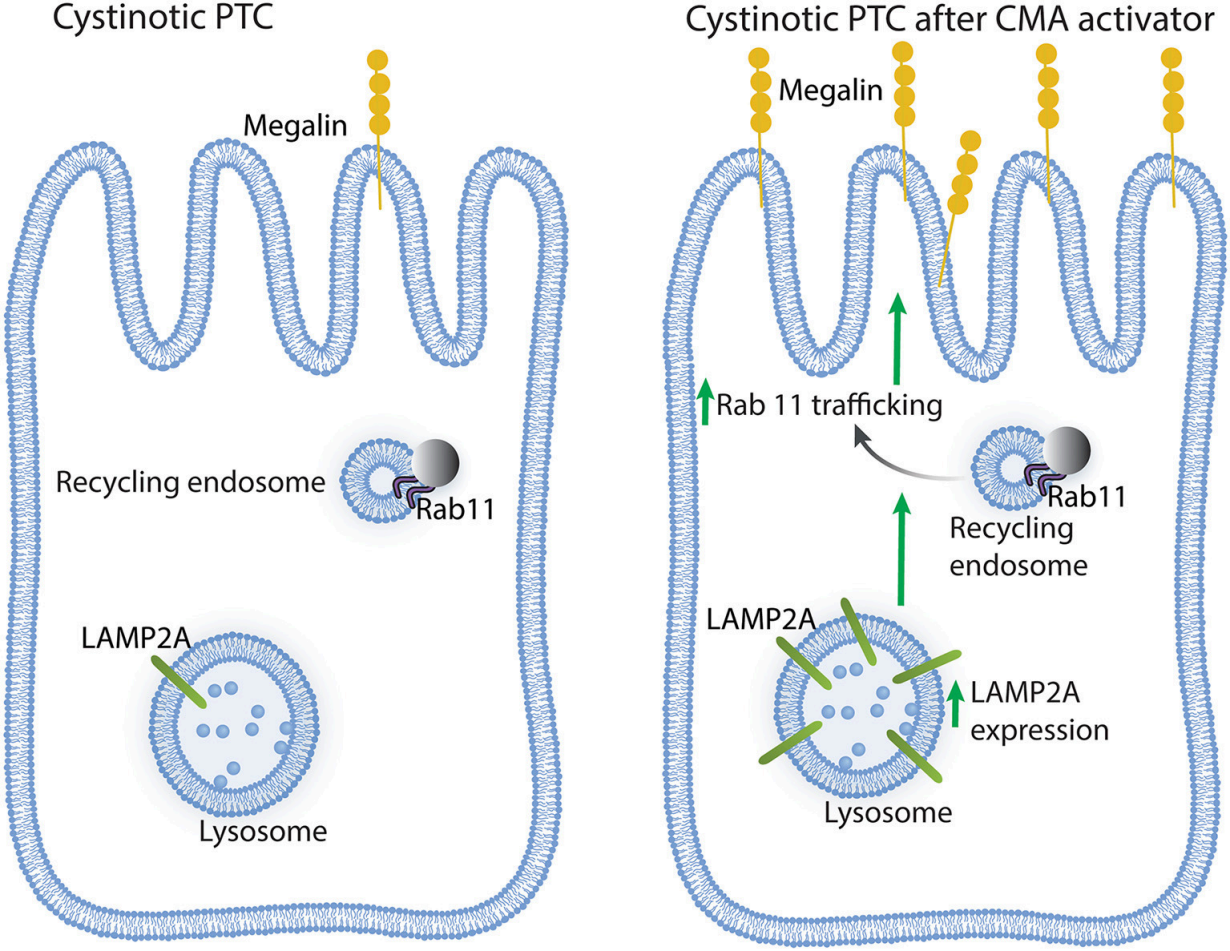
Schematic representation of the effect of chaperone-mediated autophagy (CMA) enhancers on cystinotic proximal tubule cells (PTCs). Left panel, Cystinotic PTCs are characterized by decreased localization of the CMA receptor LAMP2A at the lysosomal membrane, reduced Rab11 expression, impaired Rab11 trafficking and decreased plasma membrane localization and expression of the apical receptor megalin, a phenotype that is associated with the development of Fanconi syndrome in cystinosis. Right panel, upon treatment with CMA enhancers, cystinotic PTCs show increased LAMP2A at the lysosomal membrane, enhanced Rab11 trafficking and increased apical distribution of megalin along with increased megalin expression, suggesting that CMA upregulation has direct positive implications for PTC function.

In conclusion, CMA upregulation in cystinotic PTCs has a direct positive impact on Rab11 trafficking and megalin expression (Figure 6), whose reduced expression in cystinosis is associated with Fanconi syndrome. Thus, CMA constitutes a potential new therapeutic target, independent of lysosomal overload-reducing therapies such as cysteamine.

## Author Contributions

JZ and JH: performed experiments and organized data; JJ: supervised experiments, analyzed data, and edited manuscript; FR: performed experiments; EG and AC: contributed important reagents and edited manuscript; SC: conceived the idea, analyzed data, and wrote the manuscript.

### Conflict of Interest Statement

The authors declare that the research was conducted in the absence of any commercial or financial relationships that could be construed as a potential conflict of interest.

## Supplementary Material

Movie 1 Pseudo-TIRF microscopy analysis of GFP-Rab11 in WT HK-2 cells. Cells were transfected with GFP-Rab11 plasmid. After 48 h, cells were analyzed by TIRFM. The video was recorded for 60 s, 61 frames and 200 ms exposure. Playback is 7 fps.

Movie 2 Pseudo-TIRF microscopy analysis of GFP-Rab11 in CTNS-KO HK-2 cells. Cells were transfected with GFP-Rab11 plasmid. After 48 h, cells were analyzed by TIRFM. The video was recorded for 60 s, 61 frames and 200 ms exposure. Playback is 7 fps.

Movie 3 Pseudo-TIRF microscopy analysis of GFP-Rab11 in CTNS-KO HK-2 cells treated with QX77. Cells were transfected with GFP-Rab11 plasmid and incubated with QX77. After 48 h, cells were analyzed by TIRFM. The video was recorded for 60 s, 61 frames and 200 ms exposure. Playback is 7 fps.
